# Voltammetric Behavior, Identifying and Quantitatively Determining Iron-Based Nanoparticles, and Evaluating Their Stability in Simulated Solutions of Gastric Juice

**DOI:** 10.1155/2018/7319067

**Published:** 2018-08-01

**Authors:** N. M. Dubova, G. B. Slepchenko, I. A. Khlusov, M. S. Ostapenko, E. A. Nesterov

**Affiliations:** ^1^Physical and Analytical Chemistry Department, National Research Tomsk Polytechnic University, Tomsk, Russia; ^2^Experimental Physics Department, National Research Tomsk Polytechnic University, Tomsk, Russia; ^3^Laboratory of Immunology and Cell Biotechnologies, Immanuel Kant Baltic Federal University, Kaliningrad, Russia; ^4^Morphology and General Pathology Department, Siberian State Medical University, Tomsk, Russia; ^5^Laboratory No. 31 of the Nuclear Reactor, National Research Tomsk Polytechnic University, Tomsk, Russia

## Abstract

The electrochemical behavior of Fe_3_O_4_ nanoparticles, iron nanoparticles coated with carbon, and diazonium salts by voltammetry methods using a carbon paste electrode (CPE) has been studied. There has been developed a voltammetric method for identifying and quantifying solid-phase iron-based nanoparticles in the background electrolyte of 0.02 mole/dm^3^ Trilon B (pH 3.5) with the use of the CPE. Investigations of nanoparticles' stability with various coatings in a simulated solution of gastric juice have been carried out. Nanoparticles stability has been evaluated on the basis of determining Fe(III) ions in a simulated solution after contacting with nanoparticles within different periods of time using the method of inversion voltammetry. It has been shown that nanoparticles coated with carbon and salts of arendiazonium are the most resistant to aggressive media.

## 1. Introduction

The unique physical and chemical properties of metal nanoparticles (including iron-based nanoparticles) make it possible to use them in various sectors of the economy because of a wide range of potential use in biomedical, optical, and electronic spheres [1, 2]. In biology and medicine, nanomaterials are used, in particular, to develop facilities for directed delivery of drugs to the pathological focus of the body, as a magnetically controlled sorbent of toxins. The effect or transport of nanoparticles is often realized in an electrochemically aggressive biological environment, for example, the digestive tract (saliva and gastric and intestinal acid), which contributes to the degradation of nanoparticles with excreting ions that possess their own additional toxicity for the organism causing multiple organ pathology [3]. In connection with expanding the technological use of nanoparticles, there is growing threat of environmental pollution, in which their intake with water and food, alongside with air, is one of the main ways of getting nanoobjects into the human body and animals.

To reduce the harmful effects of nanoparticles on the human body, studies are underway to apply various protective coatings to their surface, for example, with carbon materials and organic substances [4–8]. Therefore, the problems of identifying and quantifying both the nanoparticles themselves and the products of their chemical interaction with those media in which they have been placed are acute.

In scientific literature are presented the main methods used to evaluate the physical and chemical characteristics of the functional properties of nanomaterials. Such investigations were carried out, for example, by the authors of [9–14]. To identify and determine qualitatively solid phases and various substances, highly sensitive, fairly inexpensive voltammetric methods employing an electrode with carbon paste are used [15–19]. It should be noted that, at present, there are limited data on the determination of iron-based nanoparticles using the voltammetric method with carbon paste electrode (CPE) in various sites, including biological fluids [20]. Therefore, an urgent problem is the development of methodologies for the quantitative determination and identification of iron-based nanoparticles in biological fluids using the voltammetric method.

The *work objective* is studying the electrochemical behavior of iron-based nanoparticles with various coatings using voltammetry methods, developing methods for their identification and quantitative determination, and studying stability of iron nanoparticles with various coatings in simulated solutions of gastric juice.

## 2. Experimental

In this work, there has been used a voltammetric complex for analytical measurements. The analytical voltammetric complex STA TU 4215-001-20694097-98 (Russia, Tomsk, ITM LLC) presents a compact device consisting of an electronic unit, a measuring unit with three electrochemical cells. The STA complex is completely computerized. The processing of the obtained data has been carried out using the established programs “STA.” As the indicator electrode, a carbon paste electrode (CPE), there has been used a gold-graphite electrode. The CPE is a graphite rod with the diameter of 1.5–2.0 mm pressed into a fluoroplastic case with the diameter of 5-6 mm, so that the length of the protruding part of the fluoroplastic case is 1-2 mm. For producing the carbon paste, a powder of spectral graphite of C-4 grade with a particle size of 100–200 microns has been mixed with silicone oil (1.0 g of powder and 0.5 ml of oil) and introduced into the protruding part of the fluoroplastic case. In the gold-graphite electrode, a graphite rod with the working surface diameter of 3–5 mm, impregnated with a mixture of polyethylene and paraffin in the ratio of 1 : 1, has been used. Before depositing gold films in the “in situ” mode, the graphite surface has been ground with grinding skins and a filter, and then electrochemically degreased by conducting a cathodic (at −1.0 V) and anodic (at +1.2 V) polarization with the interval of 1–2 s within 60 s.

A saturated silver chloride electrode has been used as the reference electrode.

For studies, there have been used nanopowders: sample 1, iron oxide Fe_3_O_4_ (average particle diameter 25 nm); sample 2, iron in a carbon shell (average particle diameter 5–10 nm) obtained by gas-phase synthesis at the Institute of Metal Physics, Ural Branch of the Russian Academy of Sciences (Yekaterinburg); and sample 3, iron coated with a layer of carbon and salts of arendiazonium tosylates obtained by a team of employees of the Department of Biotechnology and the Engineering School of New Production Technologies [21].

## 3. Results and Discussion

**Figure 1:**
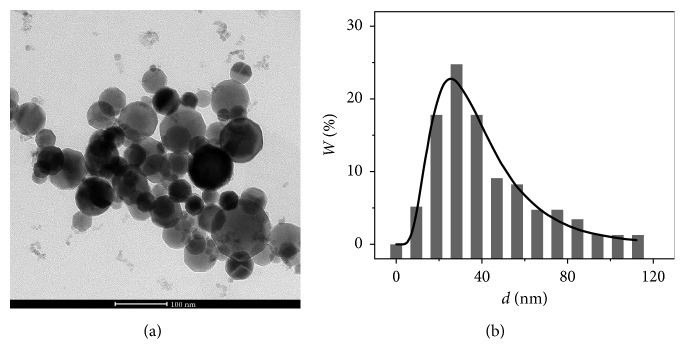
Bright field TEM image (a) and size distribution (b) of Fe_3_O_4_ nanoparticles.

Figure 1 shows the TEM image and the size distribution of Fe_3_O_4_ nanoparticles.

Transmission electron microscopy (TEM) has been carried out on the transmission microscope FEI Tecnai 20 G2 TWIN at the Laboratory of Electron Microscopy of NSTU in Novosibirsk.

Transparent electron microscopy (TEM, Tecnai 20 G2 TWIN, FEI Company, USA) has been used to study the morphology and size distribution of MNP.

According to TEM, Fe_3_O_4_ nanoparticles have a polyhedral shape with rounded edges (Figure 1(a)). The analysis of the TEM images shows a lognormal distribution in size with the average value of 25 (6–110) nm (Figure 1(b)).

Electrochemical transformations of Fe_3_O_4_ nanoparticles, carbon-coated nanoparticles, and iron nanoparticles coated with a layer of carbon and diazonium salts from the surface of the CPE under its cathodic and anodic polarization have been studied. All the studies have been carried out in the background electrolyte with 0.02 mol/dm^3^ Trilon B solution (pH 3.5).

**Figure 2:**
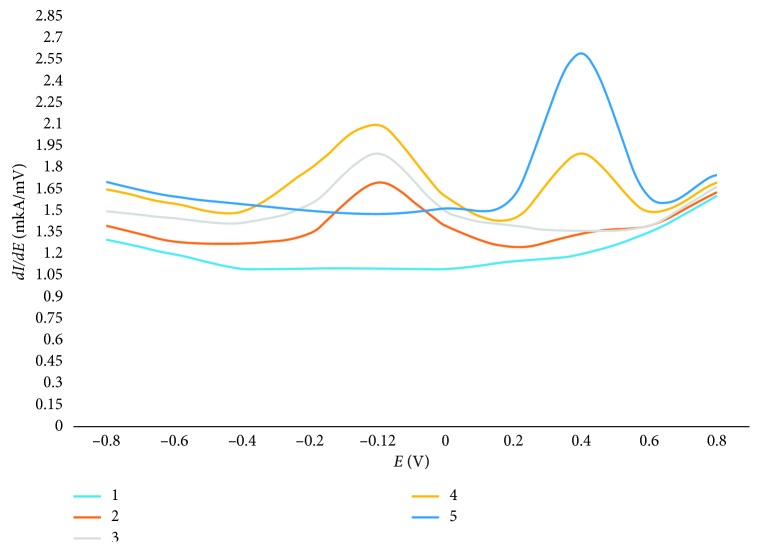
Anodic votammograms of nanoparticles from the CPE. 1, background 0.02 M Trilon B (pH 3.5); 2, Fe_3_O_4_ nanoparticles; 3, iron nanoparticles with carbon coating; 4, iron nanoparticles with carbon and diazonium salts coating; 5, diazonium salts.

The cathodic and anodic volt-ampere curves of electric transformations of Fe_3_O_4_ nanoparticles (sample 1) have been recorded in the direct current mode (1st derivative) with the potential change at the rate of 80–90 mV/s in the range from +1.0 to −1.2 V cathodic scanning, from −1.2 to +1.0 V (anodic scanning). The reproduced analytical signal of Fe_3_O_4_ nanoparticles has been observed only on the anodic voltammogram at the potential *E* = −0.12 V (Figure 2, curve 2). Since the anodic peak on the voltammogram occurs only after the cathodic polarization of the CPE, it is most likely associated with the electric oxidation of iron(II) to iron(III) from the nanoparticles to the CPE surface.

The anodic voltammogram of iron-coated carbon nanoparticles (sample 2) has also shown, as one would expect, only one anodic signal at the potential, *E*_p_ = −0.12 V (Figure 2, curve 3), coinciding with the value of the anodic peak potential of Fe_3_O_4_ nanoparticles (sample 1). The value of the anodic peak potential has not practically changed with increasing the amount of nanoparticles in the CPE. Therefore, as an analytical signal for identifying and quantitative determining solid-phase Fe_3_O_4_ nanoparticles (sample 1) and carbon-coated iron nanoparticles (sample 2), we selected the anodic peak at the potential −(0.12 ±0.01) V, which has been recorded in the anodic mode after cathodic polarization of the electrode.

To study the impact of the background electrolyte on the shape of the current-voltage curves of Fe_3_O_4_ nanoparticles (sample 1) and carbon-coated iron nanoparticles (sample 2), the CPE has been kept with different contents of nanoparticles in the background electrolyte without applying a potential to the electrode for different periods of time (1 to 30 minutes).

Keeping the CPE with nanoparticles of samples 1 and 2 in the background electrolyte within the identified time has not led to changing voltammetric curves, there has been observed a permanent value of the anodic peak potential. The value of the maximum anodic current with increasing the CPE keeping time with Fe_3_O_4_ nanoparticles in the back has not practically decreased. An insignificant decrease of the anodic current has been observed only after keeping the CPE with Fe_3_O_4_ nanoparticles in the background electrolyte within 20 minutes and longer but has not exceeded the error of the signal measurement. This fact proves the absence of chemical dissolution (degradation) of nanoparticles from the CPE surface into the background electrolyte and confirms the fact that the analytical signal is directly associated with nanoparticles electric transformations from the CPE. Besides, keeping samples 1 and 2 in the background electrolyte has not led to appearance of additional signals and the anodic peak potential shift. This proves the absence of forming some new electrically active compounds on the CPE surface with Fe_3_O_4_ nanoparticles and iron nanoparticles with carbon coating.

On the anodic current-voltage curve of iron nanoparticles coated with a layer of carbon and diazonium salts (sample 3), besides a peak (at potential −(0.12 ± 0.01)), there has been another additional anodic signal at the potential *E* = (0.45 ± 0.05) (Figure 2, curve 4) that is obviously related to electric transformations of diazonium salts. This fact is confirmed by the voltammogram shown in Figure 2, curve 5. There is presented a voltammogram of diazonium salts from the CPE. The signal value has increased with increasing the concentration of diazonium salts in the CPE. Thus, the аnalytical signal at *E*_p_ = −(0.12 ± 0.01) V of Fe_3_O_4_ nanoparticles (sample 1) with the carbon coating from the CPE surface (sample 2) on the anodic voltammogram and the analytical signal at the potential *E* = (0.45 ± 0.05) V of iron nanoparticles coated with a layer of carbon and diazonium salts (sample 3) can be used for their identification.

**Figure 3:**
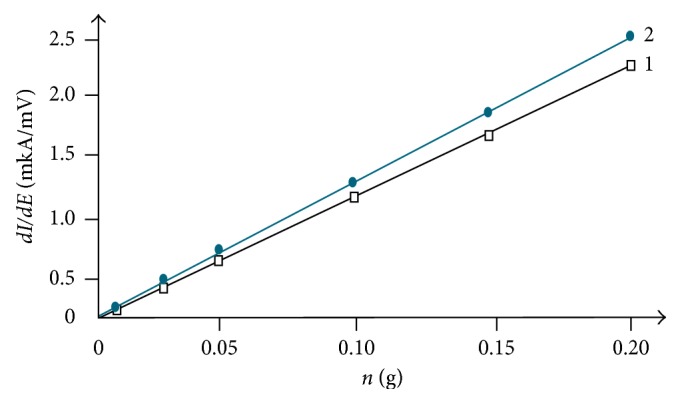
The anodic peak value dependence at *E*_p_ = −(0.12 ± 0.01) V оn the quantitative amount of nanoparticles in the CPE: 1, Fe_3_O_4_ nanoparticles; 2, iron nanoparticles in the carbon shell.

The value of anodic peaks at *E*_p_ = −(0.12 ± 0.01) V in samples 1 and 2 increased linearly with increasing the amount (weight fraction %) of nanoparticles in the CPE (Figure 3) that allows using them for the quantitative determination of the nanoparticles studied.

We estimated the quantitative number of Fe_3_O_4_ nanoparticles and iron nanoparticles in the carbon shell after contact with a physiological solution (0.5 M NaCl) for 20 minutes using the solid-phase voltammetry method in accordance with the calibration diagram constructed for the mass fraction of the Fe_3_O_4_ nanoparticle (Figure 3, curve 1) and the mass fraction of iron nanoparticles in the carbon shell (Figure 3, curve 2) in the carbon paste in the range (0.3–15)%. An estimation of the amount of nanoparticles was carried out after separation from a physiological solution by filtration, and then they were placed in CPE and analytic signals were recorded. An estimation of the correctness of the results of the voltammetric determination of nanoparticles in physiological solution (*P*=0.95; *n*=3) by the “introduced and found” method was obtained, and satisfactory convergence was obtained with an error of determination no more than 15%. These studies show the possibility of using the method of voltammetry with the CPE not only for identifying iron-based nanoparticles but also for their quantitative determination.

As it was mentioned above, in addition to the development of methods for identifying and quantifying solid-phase iron-based nanoparticles, it is of great interest to study stability of nanoparticles with various coatings (carbon and diazonium salts) in the solution of gastric juice. It has been natural to assume that when applying nanoparticles to the simulated solution of gastric juice, partial chemical dissolution will occur. We have proposed a method for studying stability (degradation) of iron nanoparticles after different periods of time of nanoparticles contacting with the model gastric juice by means of determining dissolved iron(III) in it by the method of stripping voltammetry (SV).

**Figure 4:**
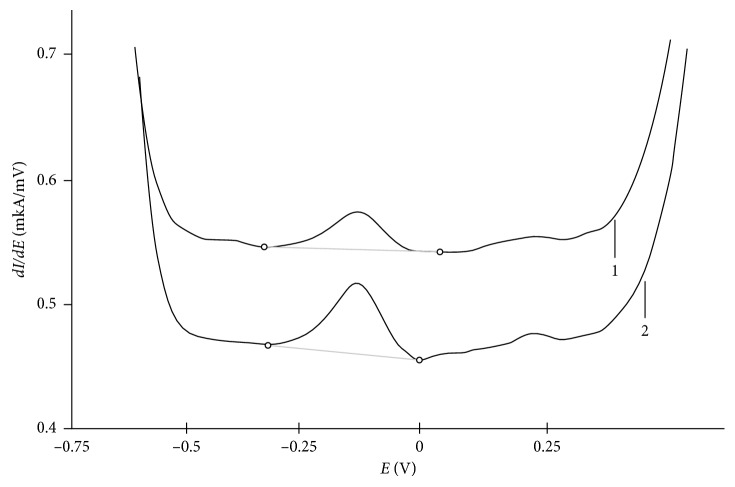
Differential voltammograms of the simulated solution of gastric juice (1) and the simulated solution with addition of iron(III) *C* = 0.1 mg/dm^3^ (2). Background is −0.02 M Trilon B gold-graphite electrode.

When studying various nanoparticles' degradation, the samples have been located in the simulated solution of gastric juice (0.1 M of HCl) and kept within different periods of time (from 1 to 22 hours). The analysis of the simulated solution of gastric juice after contacting with nanoparticles for the content of iron(III) ions has been carried out after filtering (their separation from the simulated solution of gastric juice) by the SV method with the use of the method of additives in the following conditions: the direct current mode of voltammograms (with registration of the 1st derivative); the polarizing voltage for electric accumulation *E*_e_ = −1.0 V; the speed of linear change of the potential 80 mV/s; and the background electrolyte 0.01 mol/dm^3^ Trilon B. In these conditions, the potential of the analytical signal on the gold-graphite electrode (the anode peak potential of the trilonate complex of iron oxidation) is *E*_p_ = −0.15 V.  Figure 4 shows the differential VA-curve of the simulated solution of gastric juice (curve 1) against the background Trilon B after contacting with nanoparticles.

The anodic peak potential obtained from the studied analyzed solutions (that are in contact with nanoparticles) coincides with the anodic peak potential of iron(III) ions and increases with addition of the standard solution of iron(III) (Figure 4, curve 2) that somehow proves the existence in the studied solution of iron(III), that is, chemical dissolution of nanoparticles.

**Figure 5:**
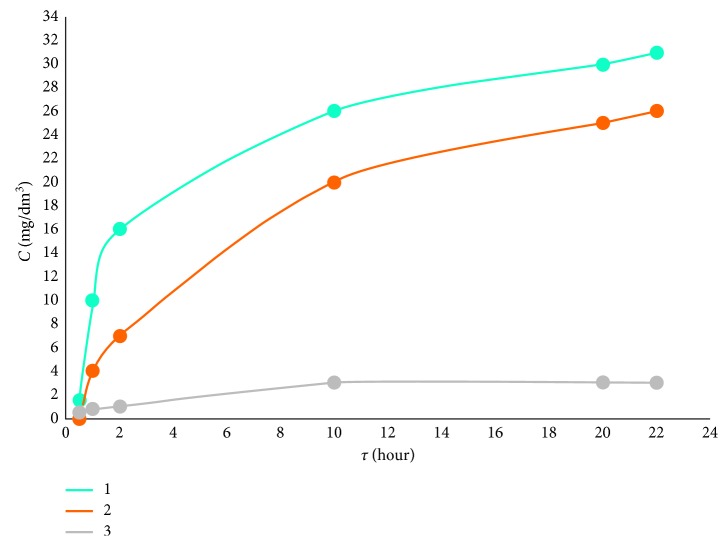
Fe(III) concentration in the simulated solution of gastric juice after keeping nanoparticles within different periods of time in 0.1 M HCl: 1, Fe_3_O_4_ nanoparticles; 2, iron nanoparticles with the carbon coating; and 3, iron nanoparticles coated with carbon and diazonium salts. Background: 0.02 M Trilon B gold-graphite electrode.

In Figure 5, curve 1, there is presented the dependence of the anodic current of iron ions value on the time of keeping nanoparticles without coating in the solution 0.1 M of HCl.

The studies carried out have shown that when Fe_3_O_4_ nanoparticles are uncoated (sample 1), the chemical dissolution of nanoparticles and the appearance of iron(III) ions in the solution of 0.1 M HCl occur almost immediately. The number of iron(III) ions transferred to the solution increases by approximately 10 times with increasing the keeping time to 120 minutes (Figure 5, curve 1).

For iron-containing nanoparticles with carbon coating (sample 2), the appearance of iron(III) ions in the test solution of gastric juice was observed only after keeping them in 0.1 M HCl solution within 1 hour (Figure 5, curve 2) which indicates greater stability of such nanoparticles in the simulated solution in comparison with iron nanoparticles without carbon coating.

When iron nanoparticles coated with a layer of carbon and diazonium salts (sample 3) and were placed in the simulated solution of gastric juice, there was observed increasing chemical stability of nanoparticles in the time intervals studied. Iron(III) ions appeared in the simulated solution after 2 hours, and their concentration in the test solution decreased by approximately 3–5 times in comparison with the previous case (Figure 5, curve 3).

Studying stability of nanoparticles with different coatings has shown that they are most resistant to aggressive media (e.g., gastric juice), and consequently, nanoparticles coated with carbon and arendiazonium salts are the least harmful to the human body.

Thus, for evaluating stability of nanoparticles with various coatings, there is applicable method of inversion voltammetry that allows in a wide range of the determined contents of iron(III) ions evaluating it with a sufficient error.

The voltammetry method of solid phases is applicable for identifying and quantitative determining solid-phase iron-based nanoparticles.

It is simple, and it does not require a large number of reagents and labor intensity and can be used in any chemical laboratory, especially at present, when there are produced analyzers with controlling and data processing. This method can be used in pharmaceutical research and in technological control of producing nanomaterials.

## 4. Conclusion

There has been studied the electrochemical behavior of Fe_3_O_4_ nanoparticles, iron-based nanoparticles with different coatings, the voltammetry method for solid phases, the voltammetry method for identifying and quantitative determining solid-phase iron-based nanoparticles, and the method for their determining in various objects including model biological objects.

By the SV method, there has been studied stability of iron-based nanoparticles in the simulated solutions of gastric juice.

## Data Availability

The data used to support the findings of this study are available from the corresponding author upon request.
